# RNA-Seq Transcriptome Analysis of Maize Inbred Carrying Nicosulfuron-Tolerant and Nicosulfuron-Susceptible Alleles

**DOI:** 10.3390/ijms16035975

**Published:** 2015-03-13

**Authors:** Xiaomin Liu, Xian Xu, Binghua Li, Xueqing Wang, Guiqi Wang, Moran Li

**Affiliations:** Institute of Cereal and Oil Crops, Hebei Academy of Agriculture and Forestry Sciences, Shijiazhuang 050035, Hebei, China; E-Mails: xianxu@hotmail.com (X.X.); binghuali@163.com (B.L.); xueqingwang@126.com (X.W.); moranli@163.com (M.L.)

**Keywords:** transcriptomics, next-generation sequencing (NGS), herbicides susceptibility, nicosulfuron, *Zea mays*

## Abstract

Postemergence applications of nicosulfuron can cause great damage to certain maize inbred lines and hybrids. Variation among different responses to nicosulfuron may be attributed to differential rates of herbicide metabolism. We employed RNA-Seq analysis to compare transcriptome responses between nicosulfuron-treated and untreated in both tolerant and susceptible maize plants. A total of 71.8 million paired end Illumina RNA-Seq reads were generated, representing the transcription of around 40,441 unique reads. About 345,171 gene ontology (GO) term assignments were conducted for the annotation in terms of biological process, cellular component and molecular function categories, and 6413 sequences with 108 enzyme commission numbers were assigned to 134 predicted Kyoto Encyclopedia of Genes and Genomes (KEGG) metabolic pathways. Digital gene expression profile (DGE) analysis using Solexa sequencing was performed within the susceptible and tolerant maize between the nicosulfuron-treated and untreated conditions, 13 genes were selected as the candidates most likely involved in herbicide metabolism, and quantitative RT-PCR validated the RNA-Seq results for eight genes. This transcriptome data may provide opportunities for the study of sulfonylurea herbicides susceptibility emergence of *Zea mays*.

## 1. Introduction

Maize (*Zea mays* L.), along with wheat and rice, are the three most widely grown food crops in the world [1]. Annual grasses, particularly goosegrass (*Eleusine indica* L.) and crabgrass (*Digitaria sanguinalis* L.) are serious weed problems in maize production in China. These vigorous weeds compete with maize for moisture, nutrients, and sunlight. Uncontrolled grass weeds can cause major yield reduction in maize [2]. Nicosulfuron developed by DuPont has been successfully used for weed control in maize. It can control a wide range of annual and perennial grasses and broadleaf weeds. Nicosulfuron has low application rates, displays low levels of acute and chronic animal toxicity, and has the widest maize safety margin, but certain maize hybrids and inbreds can be severely injured by nicosulfuron. Susceptibility of certain sweet maize hybrids to postemergence applications of nicosulfuron has been well documented in the past 20 years [3,4].

Variation among crops in response to sulfonylurea herbicides, such as nicosulfuron, mainly attributed to differential rates of herbicide metabolism. Tolerant crops detoxify herbicides more rapidly than susceptible plants [5,6]. To date, participation in herbicide detoxification metabolism has been well established for only four gene families: P450s, GSTs, ABC transporters and glycosyltransferases [7]. The cytochrome P450 family is a major family of enzymes functioning in detoxification and metabolism. P450s can mediate tolerance to all most classes of herbicides because of its group and functional diversity, broad substrate specificity, and catalytic versatility [8,9,10]. Recent studies have shown that natural tolerance in maize to a subset of sulfonylurea herbicides (nicosulfuron, rimsulfuron, primisulfuron, and thifensulfuron) is controlled by a single *CYP* gene or a group of closely-linked *CYP* genes on the short arm of chromosome 5, with resistance dominant and sensitivity recessive [11,12]. It is also known that tolerant maize metabolize nicosulfuron by hydroxylation, this hydroxylated metabolite is subsequently rapidly conjugated to glucose. Structure studies show that maize selectivity is strongly affected by substituents on the pyridine half of the molecule [13]. Glutathione *S*-transferases (GSTs) are a class of multifunctional detoxification enzymes and play an important role in the metabolism of a variety of herbicides, and plant GSTs are divided into three categories: types I, II, and III according to sequence similarity. There are a total of 42 GSTs in maize, GSTI which has been known to be a major GST component in many maize tissues, has the highest activity against many herbicides [14,15]. The increased expression and activity of GSTs has been documented as a mechanism of herbicide tolerance [16,17,18]. It is also known that ABC transporters are involved in the detoxification of xenobiotics by transporting glutathione conjugates into the vacuole, and these tonoplast transporters are inducible by certain herbicides [19,20,21]. Glycosyltransferases can conjugate a sugar molecule to a wide range of lipophilic small molecule acceptors including plant hormones, secondary metabolites, and xenobiotics such as herbicides, and the glycosylation occured at –OH, –COOH, –NH_2_, and –SH, and both *O*-glycosyltransferases and *N*-glycosyltransferases have been suggested for their roles in herbicide detoxification [22,23,24].

There is ample evidence that maize plants differ in susceptibility to sulfonylurea herbicides, but the susceptibility mechanisms are limited to biochemical, physiological and inheritance analysis [25,26,27]. Mechanisms of resistance to herbicides in plants can be roughly categorised into two classes, target-site and non-target-site resistance. Increased gene expression could be the base for both target-site and non-target-site resistance [7]. Studies showed that maize plants resistant to imidazolinone herbicides could be engineered through targeted modification of endogenous genes encoding acetohydroxyacid synthase [28]. But to date, very few cytochromes P450, glycosyltransferase, glutathione *S*-transferase and transporter genes involved in herbicide non-target-site tolerance have been identified in maize, and few genome-wide approaches to this phenomenon have been reported. Traditional method of obtaining herbicide resistant/tolerant genes has been through genetic mapping, library construction, positional cloning, which was a lengthy and expensive process [29,30,31]. RNA-Seq is the direct sequencing of transcripts by high-throughput sequencing technologies, and it has considerable advantages for providing genome-wide information, detection of novel transcripts, allele-specific expression [32]*.* It makes the obtainment of herbicide resistant/tolerant genes more fast and efficient. To date, many herbicide resistant genes were obtained in grasses such as *Eleusine indica*, *Amaranthus tuberculatus*, *Echinochloa crus-galli* using RNA-Seq technology [33,34,35]. Our objective here was to identify differentially expressed genes involved in nicosulfuron metabolism in maize, using RNA-Seq transcriptome analysis and validation experiments. These genes could serve as potential candidates to decipher sulfonylurea herbicides susceptibility formation mechanisms in maize, and provide developing strategies to improve sulfonylurea herbicides tolerance in crops.

## 2. Results and Discussion

### 2.1. Phenotypic Responses to Nicosulfuron

**Figure 1:**
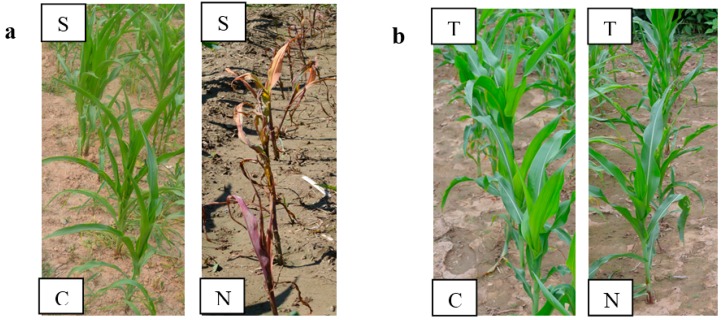
Field response test of susceptible and tolerant maize plants after application of 60 g ai ha^−1^ nicosulfuron. The photos were taken 14 days after nicosulfuron treatment. Response of untreated and nicosulfuron-treated susceptible maize plants (**a**); Response of untreated and nicosulfuron-treated tolerant maize plants (**b**). S: Nicosulfuron-susceptible maize; T: Nicosulfuron-tolerant maize; C: control untreated; N: nicosulfuron 60 g ai ha^−1^ treatment.

A field test was performed for the response analysis of susceptible and tolerant maize seedlings to 60 g ai ha^−1^ nicosulfuron when the maize at the three- to four-leaf stage. One week after nicosulfuron treatment, the 3 to 5 leaf of susceptible maize plants exhibited chlorosis, yellow, or irregular chlorotic leaf spot, the margin of some leaves shrank, and the heart-shaped leaves not normally drew, the growth of maize was inhibited severely. Two weeks later, all the susceptible maize plants were dead, but the untreated susceptible maize grew normally (Figure 1a). As for the tolerant maize, phenotypic response of nicosulfuron-treated maize was the same as the untreated, did not show any obvious damage that was caused by application of nicosulfuron (Figure 1b).

### 2.2. RNA-Seq Analysis Aligned with the Maize Reference Genome Sequence

RNA was extracted from leaves of the four samples, including the untreated/treated susceptible and untreated/treated tolerant maize, and the RNA integrity number (RIN) was 8.1, 7.9, 8.0 and 8.1, respectively. cDNA libraries developed from RNA described above were constructed and used for Illumina Genome Analyzer (HiSeq™ 2500) deep sequencing, the four libraries produced 15.4, 18.9, 18.8 and 18.7 million paired-end reads, respectively, with a single read length of about 101 bp. Q20 percentages (sequencing error rates lower than 1%) were more than 97.9%. RNA-Seq reads aligned well with the B73 reference genome, 78.9% of the filtered reads could be mapped uniquely to one location within the B73 reference genome sequence, 3.2% of the filtered reads were mapped to multiple locations. The reminding 17.9% of the reads were not mapped in the reference genome, this is mainly because the mapping uncertainty caused by paralogous gene families, low-complexity sequence and high sequence similarity between alternatively spliced isoforms of the same gene. In addition, polymorphisms, reference sequence errors and sequencing errors will also lower confidence in mappings. RNA-Seq reads do not span entire transcripts, so the transcripts from which they derived are not always uniquely determined [36].

### 2.3. Gene Functional Annotation by GO, and KEGG

**Figure 2:**
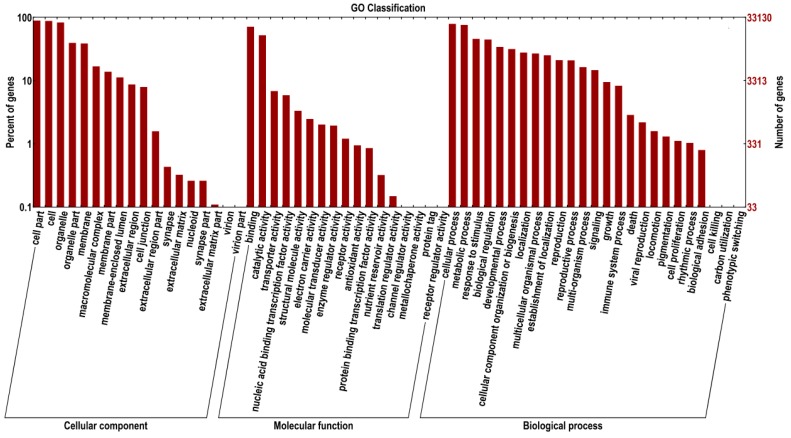
Functional annotation of genes based on gene ontology (GO) terms. GO analysis was performed at level 2 for the three main categories (biological process, cellular component and molecular function). Level 2 annotation was chosen because it greatly facilitates comparisons among sequence sets by pointing out the most significant differences. The right *y*-axis indicates the number of genes in a category. The left *y*-axis indicates the percentage of a specific category of genes in that main category.

In this study, 40,441 reads from the *Zea mays* transcriptome returned an above cut-off blast hit to the NCBI non-redundant protein database. Based on the *Zea mays* transcriptome assembly, GO terms were assigned to the annotated EST sequences using Blast2GO [37]. One or more GO terms were assigned to 33,132 (81.93%) sequences with 345,171 GO assignments in total for the biological process, cellular component and molecular function categories. The largest proportion was represented by binding (GO: 0005488; 47.65%) and catalytic activity (GO: 0003824; 22.05%) under molecular function, and cell (GO: 0005623; 21.99%), cell part (GO: 0044464; 37.63%) and organelle (GO: 0043226; 21.52%) under cellular component, and cellular process (GO: 0009987; 16.76%) and metabolic process (GO: 0008152; 15.44%) under biological process (Figure 2). Similar results were reported for *Echinochloa crus-galli*. GO terms of *Echinochloa crus-galli* revealed that the largest proportion was represented by binding (44.36%) and catalytic activity (38.66%) under molecular function, and cell (31.40%), cell part (31.40%) and organelle (25.40%) under cellular component, and the cellular process (25.16%) and metabolic process (25.05%) under biological process [34]. 

The GO terms related to herbicidal mechanisms were also analyzed: response to auxin stimulus (25), photosystem II (99), cellulose biosynthetic process (217), lipid catabolic process (95), acetyl-CoA biosynthetic process (21), carotenoid biosynthetic process (226) and acetolactate synthase activity (5). Nicosulfuron can affect sensitive plants through inhibition the activity of the enzyme acetolactate synthase (AHAS). Inhibition of AHAS always leads to the cessation of cell division and subsequent growth processes in plants. We found five sequences (GRMZM2G077215, GRMZM2G007647, GRMZM2G143008, GRMZM2G143357, GRMZM2G407044) assigned to acetolactate synthase (AHAS, EC 2.2.1.6), which is the target for nicosulfuron. GRMZM2G143008 and GRMZM2G143357 were found to be similar to *AHAS108* and *AHAS109* from *Zea mays*, while the remaining three were annotated to hypothetical proteins with unknown function. These sequences could be further investigated to determine their specific reaction to nicosulfuron stress in maize.

Functional classification and pathway assignment was performed by the Kyoto Encyclopedia of Genes and Genomes [38]. First, the 40,441 unique sequences were compared to the KEGG database using blastx with an *E*-value cutoff of <1 × 10^−5^. To identify the biological pathways that were active in the *Zea mays*, the annotated sequences were mapped to the reference canonical pathways in KEGG. In total, 6413 sequences with 108 enzyme commission (EC) numbers were mapped into 134 predicted KEGG metabolic pathways, the maps with highest unique reads representation were ribosome (Ko03010, 333 unique reads, 5.19%), followed by oxidative phosphorylation (Ko00191, 273 unique reads, 4.26%) and plant hormone signal transduction (Ko04075, 228 unique reads, 3.56%).

### 2.4. Digital Gene Expression Analysis

**Figure 3:**
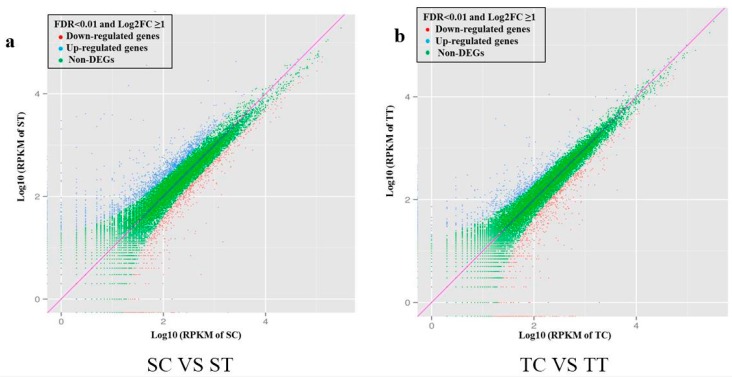
Scatter plot analysis of two sample pairs from *Zea mays*. (**a**) untreated susceptible maize (SC) and nicosulfuron-treated susceptible maize (ST); (**b**) untreated tolerant maize (TC) and nicosulfuron-treated tolerant maize (TT). RPKM (Reads Per Kilobase per Million mapped reads) were used to represent the expression levels of genes in non-nicosulfuron treated and nicosulfuron-treated libraries in susceptible and tolerant maize. Two parameters, “FDR < 0.01” and “Log2FC ≥ 1” were used as the threshold to evaluate the significance of gene expression difference. Blue and red dots represent the up- or down-regulated transcripts, respectively, and green dots indicate transcripts without significant changes under nicosulfuron treatment.

**Figure 4:**
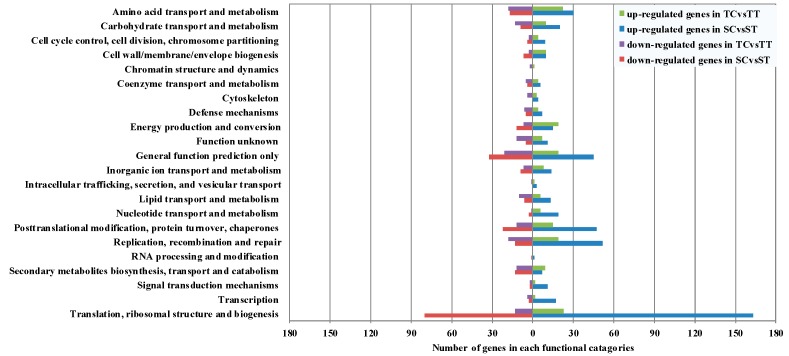
Functional classification of differentially expressed genes in the tolerant and susceptible maize. Identified differentially expressed genes (DEGs) were classified into functional categories and the number of up- or down-regulated functional categories were shown here. Transcripts without any annotation information from BLASTx program were collected into “Function unknown”.

Pairwise comparisons were made within the susceptible and tolerant maize plant between the nicosulfuron-treated and none-treated conditions. EBSeq, a free R package was used to find differentially expressed genes (DEGs) [39]. For the susceptible maize, there were 2100 genes significantly differentially expressed between untreated and nicosulfuron-treated samples. Statistical comparison revealed that 1391 genes were significantly upregulated and 709 down-regulated (Figure 3a). For the tolerant maize, there were 1398 genes significantly differentially expressed between untreated and nicosulfuron-treated samples, with 696 up-regulated and 702 down-regulated (Figure 3b). The functional classification of DEGs was further examined to investigate the pattern of transcriptome regulation that occured during nicosulfuron stress. The identified DEGs matching characterized proteins or proteins with putative functions were grouped according to functional categories. For up-regulated genes (Figure 4, right-hand side), genes encoding proteins involved in translation, ribosomal structure and biogenesis comprised the largest functional group in both susceptible and tolerant biotypes. Amino acid transport and metabolism comprised the second largest category in tolerant maize, and replication, recombination and repair comprised the second largest category in susceptible maize. Down-regulated DEGs in both nicosulfuron-treated susceptible and tolerant maize were shown in left-hand side. The down regulated genes were mainly involved in translation, ribosomal structure and biogenesis, posttranslational modification, protein turnover, chaperones in the susceptible maize, and replication, recombination and repair, amino acid transport and metabolism in the tolerant maize.

### 2.5. Searching and Validating of Potential Candidate Genes Involved in Nicosulfuron Metabolism 

Candidate genes with known roles in herbicide non-target-site and target-site resistance (ABC transporter, GST, glycosyltransferase, CYP450 and AHAS) were selected on the basis of high variation in expression between the tolerant and susceptible maize or an up-regulation more than two fold 24 h after 60 g ai ha^−1^ nicosulfuron treatment. Thirteen candidate genes were identified, including one ABC transporter, two glutathione *S*-transferase, one glycosyltransferase, seven cytochromes P450 and two acetolactate synthase (Table 1). 

**Table 1:** Differentially expressed candidate genes between nicosulfuron-susceptible (S) and tolerant (T) *Zea mays* using RNA-Seq. SC: untreated susceptible maize; ST: nicosulfuron-treated susceptible maize; TC: untreated tolerant maize; TT: nicosulfuron-treated tolerant maize.

Sequence ID	Annotation	fpkm			
		SC	ST	TC	TT
GRMZM2G119345	ABC transporter protein	12.6	28.3	27.0	40.7
GRMZM2G116273	glutathione *S*-transferase gene GST1	558.2	805.0	711.0	1019.3
GRMZM2G330635	glutathione *S*-transferase gene GSTU6	61.7	99.2	156.9	157.4
GRMZM2G051367	Glycosyltransferase	8.9	18.1	22.6	24.8
GRMZM2G370745	cytochrome P450 monooxygenase, CYP72A28	18.8	28.0	26.2	64.7
GRMZM2G022947	cytochrome P450 monooxygenase, CYP727A4	12.7	25.3	19.2	25.9
GRMZM2G129860	cytochrome P450 monooxygenase, CYP72A5	3.0	7.7	24.6	25.2
AC217947.4_FG002	cytochrome P450 monooxygenase	3.7	9.6	31.0	55.1
GRMZM2G093286	cytochrome P450 monooxygenase, CYP78A55	5.6	11.3	11.7	37.3
GRMZM2G063756	cytochrome P450 monooxygenase, CYP71C3v2	269.5	395.6	1131.4	1380.3
GRMZM2G090432	cytochrome P450 monooxygenase, CYP81A9	0	0.1	57.5	68.0
GRMZM2G143357	acetolactate synthase, AHAS109	4.2	24.5	5.5	6.3
GRMZM2G143008	acetolactate synthase, AHAS108	24.9	81.5	24.5	34.1

Cytochromes P450, glutathione-*S*-transferase, glycosyltransferase and transporter genes can be involved in herbicide non-target-site resistance via enhanced expression [40]. Among these mechanisms, the oxidization of herbicides by endogenous cytochrome P450 monooxygenase is thought to be a major pathway in plants [10]. In our study, We observed that there were seven cytochromes P450 candidate genes up-regulated in both tolerant and susceptible maize 24 h after nicosulfuron treatment, including CYP71C, CYP72A, CYP78A and CYP81A families. The expression level of these genes in the tolerant maize was much higher than the susceptible, especially for the *CYP71C3v2* and *CYP81A9*. The *CYP81A9* gene was once demonstrated to be closely related to nicosulfuron detoxification in maize [41]. Some cytochromes P450 in families CYP71C, CYP72A and CYP81A were once demonstrated to involve in herbicide metabolism directly in the other crops, such as wheat *CYP71C6v1*, rice *CYP72A31* and *CYP81A6* [8,42,43]. Glutathione *S*-transferase gene *GST1* and *GSTU6* were also found to be up-regulated in maize after nicosulfuron treatment. Compared with the susceptible maize, the expression level of *GST1* and *GSTU6* was induced more by nicosulfuron in the tolerant. And it was once demonstrated that transgenic tobacco plants expressing maize *GST1* developed for enhanced detoxification of herbicide alachlor [44]. The ABC transporter gene *ZmMRP1* in maize and glycosyltransferases gene *Os01g31370* in rice are involved in regulation of plant response to metolachlor and atrazine, respectively [20,45]. In our study, the ABC transporter gene (GRMZM2G119345) and glycosyltransferases gene (GRMZM2G051367) were also found to involve in the metabolism of nicosulfuron. The RNA-Seq data also showed that expression level of *AHAS108* was much higher than *AHAS109* in both susceptible and tolerant maize, and when treated with nicosulfuron, the expression level of the *AHAS* gene in susceptible maize increased more significantly than the tolerant maize. Differential gene expression within the *AHAS* gene family is known in *Nicotiana tabacum*, *Brassica napus* and *Glycine max* [46,47]. Differences in *AHAS* gene expression levels exist among populations, and these variations will exert an influence on the evolution of herbicide tolerance [48]. 

Our data support the hypothesis that herbicide response in plants is driven by differential expression of a set of genes. The candidate genes identified are potentially useful for developing molecular assays to help detecting nicosulfuron tolerance or susceptibility in maize. As gene expression regulation also involves post-transcriptional steps, the possible direct role of these non-target-site and target-site candidate genes will be further investigated, and functional analysis of these genes will be conducted in future to determine their relationship with nicosulfuron tolerance in maize.

**Figure 5:**
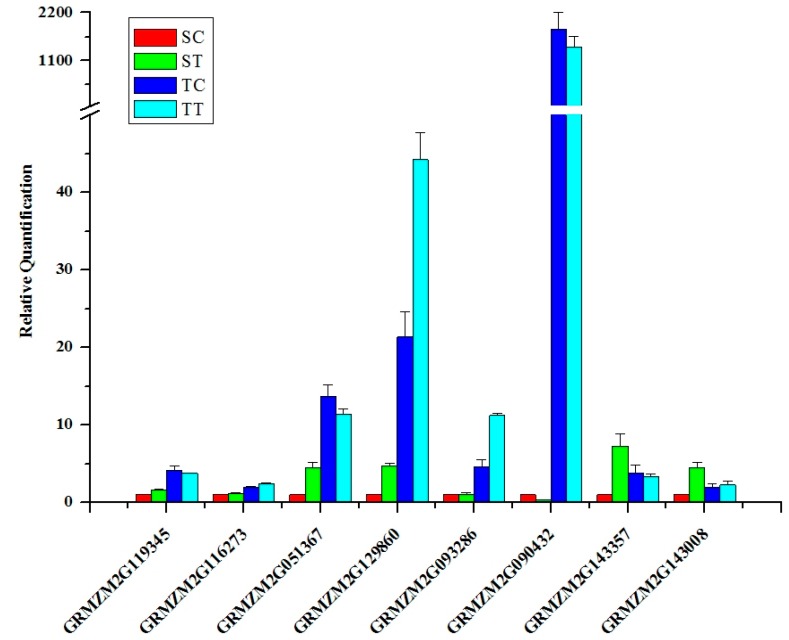
Real-time RT-PCR analysis for eight differentially expressed genes in nicosulfuron-tolerant and nicosulfuron-susceptible *Zea mays*. Real-time RT-PCR was carried out on three independent biological replicates each containing three technical replicates. The relative quantification of each transcript was normalized against β-actin. SC: untreated susceptible maize; ST: nicosulfuron-treated susceptible maize; TC: untreated tolerant maize; TT: nicosulfuron-treated tolerant maize.

To confirm the accuracy and reproducibility of the Illumina RNA-Seq results, a few genes with a differential change in expression were selected from maize DEGs for real-time RT-PCR analysis. β-actin was used as reference gene for data normalization according to Vanessa Galli *et al.* [49]. The Real-time RT-PCR results are shown in Figure 5. The expression patterns of all detected genes show the same trend using RT-PCR and the Solexa-sequencing method.

## 3. Experimental Section 

### 3.1. Plant Material and RNA Extraction

The response of tolerant and susceptible maize plants to 60 g ai ha^−1^ nicosulfuron was examined in the field experiment. The maize plants were collected from Institute of Cereal and Oil Crops, Hebei Academy of Agriculture and Forestry Sciences, China. Commercial formulations of nicosulfuron were applied when the maize at the three- to four-leaf stage, and the phenotypic responses of the treated and untreated maize were observed until 28 days after the application of nicosulfuron.

For the RNA extraction, plants were grown under greenhouse conditions (27 °C/24 °C day/night and 16 h/8 h light/dark) with three plants per pot. Two weeks later, the susceptible and tolerant maize seedlings were treated with 60 g ai ha^−1^ nicosulfuron. After 24 h of spraying, the treated and untreated leaves of each maize plant were harvested, and then were placed in a mortar, mixed with liquid nitrogen and fully grounded. Total RNA was extracted from the samples using Trizol reagent (Invitrogen, San Diego, CA, USA) according to the manufacturer’s instructions. The quantity and quality of RNA samples were assessed using 1% agarose gel and examined with a Nanodrop 1000 spectrophotometer (Nanodrop, Wilmington, DE, USA). The RIN (RNA Integrity Number) values of the samples were assessed using an Agilent 2100 Bioanalyzer (Agilent Technologies, Santa Clara, CA, USA).

### 3.2. cDNA Library Construction and RNA-Seq

High quality total RNA (5 µg, 100 ng/μL) samples were sent to the Biomarker Biotechnology Corporation (Beijing, China) for RNA-Seq library preparation. mRNA was enriched and purified with Oligo(dT)-rich magnetic beads and then broken into short fragments. Taking these cleaved mRNA fragments as templates, first-strand cDNA and second-strand cDNA were synthesized. The resulting cDNAs were then subjected to end-repair and phosphorylation using T_4_ DNA polymerase and Klenow DNA polymerase. After that, an “A” base was overhung at the 3' ends of the repaired cDNA fragments and Illumina paired-end solexa adapters were subsequently ligated to these cDNA fragments to distinguish the different sequencing samples. To select a size range of templates for downstream enrichment, the products of the ligation reaction were purified and selected on a 2% agarose gel. Next, PCR amplification was performed to enrich the purified cDNA template. Finally, the four libraries of both nicosulfuron treated/untreated tolerant and susceptible maize described above were sequenced using an Illumina HiSeq™ 2500 (Biomarker Technologies Corporation, Beijing, China).

### 3.3. RNA-Seq Reads Mapping and Transcript Assembly

After removing those with only adaptor and unknown nucleotides larger than 5%, or those that were of low quality, the clean reads were filtered from the raw reads. Cleaned RNA-Seq reads were then mapped to the maize B73 reference genome version 2 (http://ftp.maizesequence.org/) using Bowtie version 0.12.7 [50] and TopHat version 1.4.1 [51].

The SAM (Sequence Alignment/Map) files generated by Tophat were provided as input to the software Cufflinks [52], which assembles the alignments in the SAM file into transfrags. Cufflinks does this assembly independently of the existing gene annotations. Cufflinks constructs a minimum set of transcripts that best describes the RNA-Seq reads. The unit of measurement used by Cufflinks to estimate transcript abundance is Fragments Per Kilobase of exon per Million fragments mapped (FPKM). Cufflinks statistical model probabilistically assigns reads to the assembled isoforms.

### 3.4. Genes Annotation

Unique reads were aligned to a series of protein databases using blastx (*E*-value ≤ 10^−5^), including the NCBI non-redundant (Nr), the Swiss-Prot, the Trembl, the Kyoto Encyclopedia of Genes and Genomes (KEGG) and Gene ontology (GO) databases. To evaluate the coverage depth, all usable reads were realigned to each unique reads using SOAPaligner (http://soap.genomics.org.cn/soapaligner.html), then normalized into RPKM value (reads per kb per million reads). After that, reads abundance differences between the samples were calculated based on the ratio of the RPKM values, and the False Discovery Rate (FDR) control method was used to identify the threshold of the *p* value in multiple tests in order to compute the significance of the differences in transcript abundance. Here, only unique reads with an absolute value of log2 Ratio ≥1 and a FDR significance score <0.01 were used for subsequent analysis.

### 3.5. Gene Validation and Expression Analysis

Some of the nicosulfuron-tolerant unique reads were subjected to real-time quantitative PCR (q-PCR) with specific primers identified by Primer Premier software (Premier Biosoft International, Palo Alto, CA, USA). The primers of selected genes are listed in Table 2. cDNA synthesis and q-PCR were performed as described. The cDNAs were amplified by RT-qPCR in a final volume of 20 µL containing 1 μL cDNA, 10 μL 2× qPCR Master Mix, and 10 µmol of each primer. Amplification was standardized in a 7500 Real-time Fast thermal cycler (Applied Biosystems, Foster City, CA, USA) using the following conditions: 50 °C for 20 s, 95 °C for 10 min followed by 45 cycles of 3 min at 94 °C, 15 s at 94 °C, 15 s at 58 °C and 20 s at 72 °C. The PCR products for each primer set were subjected to melting curve analysis to verify the presence of primer dimers or non-specific amplicons. The melting curve analysis ranged from 60 to 95 °C, with an increase in the temperature stepwise by 1%. The actin gene was used as the internal control for normalization of gene expression. Three independent biological replicates and three technical replicates of each biological replicates for each sample were analyzed in q-PCR analysis to ensure reproducibility and reliability. 

**Table 2:** List of primers used for the Real-time RT-PCR.

Sequence ID	Forward Primer	Reverse Primer	Target Size bp
GRMZM2G119345	AGGGTAGGATTCTGATGTTC	TGCTGATACTTCGGTCTGTTT	73
GRMZM2G116273	GGGGAACCACCGACCAGAAAG	GCGTAGGGCGTAGCAAACAGG	172
GRMZM2G051367	CGTTGCCTCCATCGCTTACTG	TGCCTGGTTCATTGGTCTCCC	276
GRMZM2G129860	CGCCATCCTACACCCACG	TATGCGGTCAGTAACGAAA	138
GRMZM2G093286	GGTTCGTGTTCGGCAAGGAG	GGGAAGTAGTCGCACAGGTT	136
GRMZM2G090432	ACCACCCAACAGCCAAACCA	CCCAGGAGGTAGTGGAGCAA	102
GRMZM2G143357	TGCTAAAGGGTTCAACATTCC	ACAGTCCTGCCATCACCATCC	195
GRMZM2G143008	TTCTTCCTCGCCTCCTCTGGTC	ACAAAGCGTCGCAACTCCTCAC	248
β-actin	CATGGAGAACTGGCATCACACCTT	CTGCGTCATTTTCTCTCTGTTGGC	118

## 4. Conclusions 

The transcriptomes of the tolerant and susceptible maize inbred in response to nicosulfuron were surveyed using the RNA-Seq technology. The dataset generated in this study provides a significant resource for further molecular studies of the herbicide metabolism in *Zea mays*. Using this approach, thirteen genes were selected as the candidates most likely involved in nicosulfuron metabolism, the expression level of these genes in the tolerant maize was much higher than the susceptible maize. The transcriptome data from this study will facilitate further understanding of the nicosulfuron susceptibility emergence of *Zea mays*.
